# Reversal of cerebral radiation necrosis with bevacizumab treatment in 17 Chinese patients

**DOI:** 10.1186/2047-783X-17-25

**Published:** 2012-08-23

**Authors:** Yang Wang, Li Pan, Xiaofang Sheng, Yin Mao, Yu Yao, Enmin Wang, Nan Zhang, Jiazhong Dai

**Affiliations:** 1 Department of Radiation Oncology, Huashan Hospital, Fudan University, Shanghai, 200235, China; 2 Department of Neurosurgery, Huashan Hospital, Fudan University, Shanghai, 200235, China

**Keywords:** Bevacizumab, Cerebral capillaries, Radiation necrosis, Vascular endothelial growth factor

## Abstract

**Background:**

Bevacizumab has been suggested as a new treatment modality for cerebral radiation necrosis due to its ability to block the effects of vascular endothelial growth factor (VEGF) in leakage-prone capillaries, though its use still remains controversial in clinical practice.

**Methods:**

The use of bevacizumab in 17 patients with symptomatic cerebral radiation necrosis poorly controlled with dexamethasone steroid treatments was examined between March 2010 and January 2012. Bevacizumab therapy was administered for a minimum of two cycles (7.5 mg/kg, at two-week interval) with a median of four bevacizumab injections. Changes in bi-dimensional measurements of the largest radiation necrosis lesions were observed by gadolinium-enhanced and T2-weighted magnetic resonance imaging (MRI). Additionally, dexamethasone dosage, Karnofsky performance status (KPS), adverse event occurrence and associated clinical outcomes were recorded for each patient.

**Results:**

MRI analysis revealed that the average reduction was 54.9% and 48.4% in post-gadolinium and T2-weighted sequence analysis, respectively. Significant clinical neurological improvements were expressed in 10 patients according to KPS values. Dexamethasone reduction was achieved four weeks after initiation of bevacizumab in all patients, with four patients successfully discontinuing dexamethasone treatment. Mild to moderate bevacizumab-related adverse events, such as fatigue, proteinuria and hypertension were observed in three patients. Upon follow-up at 4 to 12 months, 10 patients showed clinical improvement, and 7 patient deaths occurred from tumor progression (5 patients), recurrent necrosis (1 patient), and uncontrolled necrosis-induced edema (1 patient).

**Conclusions:**

These findings suggest bevacizumab as a promising treatment for cerebral radiation necrosis induced by common radiation therapies, including external beam radiotherapy (EBRT), stereotactic radiosurgery (SRS), and fractionated stereotactic radiotherapy (FSRT).

## Background

Cerebral radiation necrosis, typically manifesting as a necrotic white matter lesion, is one of the most dreaded toxicities associated with radiation therapies targeting brain tissues. The condition often appears three or more months after treatment, with treatment volume and radiation dosage being the two most important predictors of occurrence and severity. Clinical appearance of radiation necrosis has historically been treated by application of corticosteroids; however, relapse frequently occurs upon steroid discontinuation. Also, poor patient response to conventional steroid dosages has been previously observed, including adverse effects such as behavioral changes, altered sleep patterns and changes in appetite. Recent research suggests that therapeutic anticoagulation and hyperbaric oxygen therapy may provide some relief of these symptoms, but the efficacy of these treatments is still controversial [1]. Alternatively, surgical decompression of radiation necrosis lesions can provide a beneficial palliative effect; however, this treatment does not reverse the necrotic process in the majority of patients [2].

Bevacizumab is a humanized murine monoclonal antibody that is used directly against vascular endothelial growth factor (VEGF), it has been used in the treatment of a variety of solid tumors [3]. A growing number of researchers have published positive findings using bevacizumab (Avastin, Genentech, San Francisco, CA, USA) as a treatment strategy for cerebral radiation necrosis due to its ability to block the effects of VEGF in leakage-prone capillaries [3,4]. A randomized controlled clinical trial further demonstrated class I evidence of the efficacy of bevacizumab treatment for progressive radiation necrosis [5]. Despite these positive results, there remains controversy pertaining to the efficacy of this treatment, with some researchers claiming that bevacizumab treatment reverses cerebral radiation necrosis effectively [6], while others claim that such treatment increases the risk for over-pruning of blood vessels that could potentially exacerbate cerebral radiation necrosis [7].

The pathogenesis of cerebral radiation necrosis is thought to be initiated by endothelial cell dysfunction associated with the release of VEGF, a substance capable of disrupting the blood brain barrier [8]. Previously, VEGF was referred to as a “vascular permeability factor” with the potential to cause capillary endothelial leakage in cerebral tissues [9]. Thus, VEGF is likely to play a key role in radiation necrosis development [10]. When it is released in the presence of hypoxia and necrosis, increased permeability of the blood–brain barrier and subsequently increased edema result [11]. The breakdown of the blood brain barrier leads to significant edema formation that is readily apparent through T2-weighted MRI, fluid attenuated inversion recovery (FLAIR) images, and T1-weighted gadolinium-enhanced MRI. Given its association with radiation necrosis and blood–brain barrier dysfunction, VEGF may become a logical putative therapeutic target for the reversal of edema achieved by preventing VEGF from reaching its capillary targets [3].

The current study reports on the findings observed at a single clinical facility in China resulting from bevacizumab treatment in patients with cerebral radiation necrosis not well controlled with dexamethasone treatments.

## Methods

A total of 17 symptomatic cerebral radiation necrosis patients aged 13 to 71 years (median: 48), evidencing no improvement upon treatment with the steroid dexamethasone, were selected for bevacizumab therapy treatment at Shanghai Gamma Knife Hospital between March 2010 and January 2012. Full patient profiles are listed in Table 1. Hematology analysis, biochemistry tests and patient history were analyzed in each case, resulting in exclusion based on occurrence of major surgical procedures or traumatic injuries within the past 28 days, uncontrolled hypertension, cardiovascular or cerebrovascular disease within the last six months, coagulopathy with an increased risk of bleeding, proteinuria or renal dysfunction, and non-healing wounds or ulcers. MRI imaging was applied prior to bevacizumab treatment in order to exclude cerebral hemorrhaging. All data were prospectively recorded and analyzed. Approval was obtained from the Ethics Committee of Shanghai Gamma-knife Hospital. Each patient provided written informed consent prior to treatment and inclusion in the present study.

**Table 1:** Patient profiles for 17 patients presenting symptomatic radiation necrosis

**Pt. No**	**Age (y)**	**Sex**	**Underlying disease**	**Radiation treatment**	**Radiation necrosis site**	**Last RT to study entry (mo)**	**% Change in Gadolinium**	**% Change in T2W**	**Radiologic follow-up**	KPS^*#^	Steroids^**#^**(mg)**
1	70	M	Colon cancer	SRS 17 Gy	Left temporal	6	0	0	4	30 → 30	15 → 12.5
2	13	F	Anaplastic Astrocytoma	EBRT 56 Gy	Right thalamus	18	75	60	9	50 → 70	10 → 0
3	71	M	Colon cancer	EBRT 36 Gy	Left frontal	4	65	10	6	40 → 80	15 → 5
4	52	F	meningioma	EBRT 60 Gy	Right frontal	180	80	60	8	60 → 90	10 → 2.5
5	71	M	Lung cancer	FSRT 31.5 Gy/3 fx	Left occipital	7	87	78	8	50 → 90	15 → 0
6	48	M	Glioblastoma	EBRT 60 Gy	Left frontal	7	80	75	5	50 → 70	10 → 2.5
7	32	F	Anaplastic oligodendroglioma	EBRT 60 Gy	Right frontal	40	50	45	4	50 → 70	15 → 10
8	67	F	Colon cancer	1.EBRT 39 Gy /13 fx	Right frontal	1	52	78	4	50 → 90	15 → 0
				2.SRS 16 Gy							
9	23	M	AVM	SRS 16 Gy	Left basal ganglia	21	50	40	3	70 → 80	10 → 5
10	26	M	Fibrous dysplasia of bone	EBRT 70 Gy	Brain stem	132	65	55	4	50 → 70	17.5 → 5
11	62	M	Glioblastoma	EBRT 60 Gy	Left frontal	9	73	61	8	30 → 50	15 → 5
12	65	M	Glioblastoma	EBRT 60 Gy	Right frontal	4	30	45	6	60 → 70	10 → 2.5
13	41	M	Glioblastoma	FSRT 30 Gy/6 fx	Left basal ganglia	15	60	50	8	50 → 90	15 → 2.5
14	45	M	Glioblastoma	FSRT 25.2 Gy/6 fx	Right thalamus	4	33	76	6	60 → 90	15 → 2.5
15	46	M	Lung cancer	1.EBRT 30 Gy/10 fx	Left occipital	5	50	30	8	60 → 80	12.5 → 5
				2.SRS 16 Gy							
16	34	M	Glioblastoma	EBRT 60 Gy	Right temporal	5	75	54	4	60 → 80	15 → 5
17	55	M	Glioblastoma	EBRT 56 Gy	Right temporal	6	60	65	8	40 → 60	10 → 0

Abbreviations: AVM, arteriovenous malformation; EBRT, external beam radiation therapy; F, female; FSRT, fractionated stereotactic radiotherapy; KPS, Karnofsky performance status; M, male; RT, radiation treatment; SRS, stereotactic radiosurgery; T2W, T2-weighted MRI.

Note: ^*^ Karnofsky performance scores were assessed immediately prior to treatment and at four weeks after treatment initiation.

^**^ Amounts of dexamethasone (mg/day) immediately prior to the treatment and four weeks after treatment initiation.

^#^ pretreatment → post two injections of bevacizumab.

Pathological examination revealed various histological types in original tumor tissues, including glioblastoma, anaplastic astrocytoma, anaplastic oligodendroglioma, arteriovenous malformation (AVM), metastatic brain tumor, meningioma and fibrous dysplasia of bone. All patients had previously undergone conventional radiation therapy using external beam radiation therapy (EBRT), stereotactic radiosurgery (SRS), or cyberknife treatment system based fractionated stereotactic radiotherapy (FSRT) either with or without concurrent chemotherapy. Of the 17 included patients, 2 patients had received prior SRS, 3 patients had received cyberknife treatment system based FSRT and 10 patients had received EBRT only. The two patients previously treated with conventional whole brain radiotherapy received a second course of radiotherapy with SRS due to tumor recurrence.

Diagnosis of cerebral radiation necrosis was based on the clinical presentations and radiologic imaging findings, including findings from magnetic resonance imaging (MRI), magnetic resonance spectroscopy (MRS), perfusion MRI, and ^18^F-2-fluro-D-deoxy-glucose-positron emission tomography (FDG-PET). MRI images demonstrated an enhanced irradiated field with increased T2-weighted signals in the surrounding brain parenchyma. Radiation necrosis in MRS analysis was characterized by decreased peaks in Cho, NAA and Cr, low Cho/Cr values, and elevated Lip-Lac/Cho values compared with healthy brain tissues [12]. FDG-PET scans showed no uptake of radionuclides, thus ruling out tumor progression. Notably, imaging scans clearly evidencing radiation necrosis cannot exclude the possibility of the presence of a small number of living tumor cells in or surrounding the lesion. Thus, such determination methods relying primarily on imaging indicate only that radiation necrosis is the primary cause of radiographic enhancement and perilesional edema.

All 17 patients were treated with the steroid dexamethasone (median dose of 5 mg, three times daily, two to four weeks) with no significant improvement in symptoms or MRI findings. All patients underwent MRI imaging prior to bevacizumab treatment in order to establish a baseline imaging profile. Similarly, all but two patients underwent MRS. Intravenous bevacizumab treatment was then started at 7.5 mg/kg for a minimum of two treatments with a two-week interval between treatments, with individual treatments administered over the course of 90 minutes. Primary response assessment was performed two weeks after the second dose of bevacizumab and every eight weeks thereafter. Notably, patients received only a single round of bevacizumab therapy, and no second round treatments were administered to treat recurrent radiation necrosis or uncontrolled edema.

Bi-dimensional measurement was defined as the product of the longest diameter and its longest perpendicular diameter per the MacDonald criteria [13]. These measurement were recorded for the largest radiation necrosis lesions observed in both gadolinium-enhanced and T2-weighted sequences [13]. Measurements were then calculated and presented as a percentage change from baseline MRI profiles. Other data, including dexamethasone dose, Karnofsky performance status (KPS), adverse event occurrence and clinical outcomes, were also recorded. Toxicity was graded according the National Cancer Institute Common Toxicity Criteria (Version 3.0).

## Results

The median length of bevacizumab therapy was seven months, and all patients were treated with a median of four bevacizumab injections. MRI imaging revealed a primary response assessment showing an average reduction of 54.9% and 48.4% in post-gadolinium and T2-weighted scans, respectively. These findings indicated a good and rapid response to bevacizumab treatment for reversal of cerebral radiation necrosis. All patients, with only one exception, showed significant clinical improvement in terms of neurologic symptoms expressed by KPS. KPS elevation was seen in 16 patients, with an increase of 24.7 in average scores. Only a single patient exhibited no change in KPS score. Additionally, a single patient with metastatic brain cancer originating from colon cancer did not show significant clinical improvement or reduction of edema area by MRI analysis even after four cycles of bevacizumab treatment. No worsening symptoms were observed in any patients during treatment with low dose dexamethasone until two months after the last cycle of bevacizumab treatment.

The median length of follow-up was 6 months (4 to 12 months) after the last dose of bevacizumab. Of the 17 included patients, 10 showed clinical improvement at the end of the follow-up period. The other seven patients died from tumor progression (five patients), recurrent radiation necrosis four months after bevacizumab treatment (one patient), and uncontrolled cerebral edema due to radiation necrosis (one patient).

**Figure 1:**
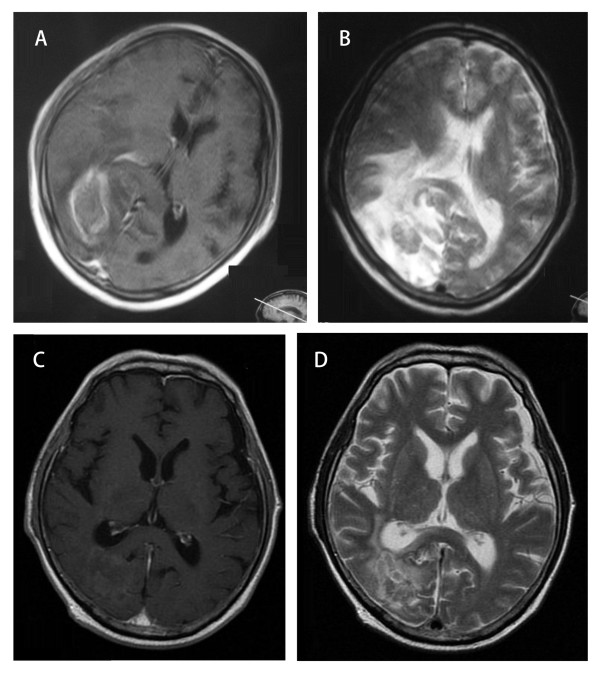
**MRI images before (A, B) and after (C, D) bevacizumab treatment in Patient No. 5.** Patient No. 5 (Table 1) received two cycles of bevacizumab (7.5 mg/kg, two-week intervals) for brain metastatic tumor from non-small cell lung carcinoma cancer. The patient also received cyberknife treatment system based FSRT with 31.5 Gy in three fractions seven months prior to treatment.

Notably, T1-weighted gadolinium-enhanced and T2-weighted MRI images before (Figure 1A, B) and after (Figure 1C, D) bevacizumab treatment indicate that bevacizumab treatment decrease perilesional edema caused by radiation necrosis. The patient shown received two cycles of bevacizumab (7.5 mg/kg, at two-week intervals) for a brain metastatic tumor from non-small cell lung carcinoma cancer. The patient also received cyberknife treatment system based FSRT with 31.5 Gy in three fractions seven months prior to treatment.

At the time of registration, dexamethasone dosages ranged from 10 to 17.5 mg/day (median = 15 mg) before bevacizumab treatment. Dosage reduction was achieved in all patients four weeks after initiation of bevacizumab treatment. A total of four patients successfully discontinued dexamethasone therapy at that time.

Bevacizumab-related adverse events of grade II or less occurred in three patients. Hypertension, subsequently treated by oral medication, occurred in one patient after the second bevacizumab injection. A single patient reported temporary fatigue after the first bevacizumab injection, which later resolved spontaneously. Proteinuria (++) accompanied by positive fecal occult blood testing (++) was observed after two injections in the patient with brain metastasis of colon cancer, though the signs were not found aggravated in the remaining follow-up studies. No indications of previously reported severe adverse events related to bevacizumab treatment, including gastrointestinal perforation, cerebral hemorrhage, arterial thromboembolic event and infusion reaction were observed in the current study.

## Discussion

Bevacizumab has the ability to reverse radiation necrosis in patients previously treated with conventional radiation therapy for brain cancer. The current findings suggest that bevacizumab treatment represents no severe risks for patients, unlike some previous studies. The potential beneficial effects of bevacizumab treatment on radiation necrosis were first reported by Gonzalez *et al.*[3] in a retrospective analysis published in 2007 in which all eight patients treated with bevacizumab showed radiographic improvement with an average of 44% reduction in gadolinium-enhanced MRI studies and 59.75% reduction in FLAIR MRI studies. The current study reports similarly large reductions in necrotic tissues after bevacizumab treatment. In the current study, average dexamethasone dosages prior to bevacizumab treatment were 10.5 mg/day. These treatments were gradually reduced to an average of 3 mg/day. This remarkable reduction was thought to be the result of restoration of the blood brain barrier by bevacizumab action. In 2008, Wong *et al.* reported a significant improvement in patients with neurocognitive deficits after bevacizumab therapy, as observed through imaging [6]. In a randomized double-blind placebo-controlled trial of bevacizumab treatment in 14 patients with radiation necrosis of the central nervous system, bevacizumab (7.5 mg/kg) or saline was administered intravenously at three-week intervals in the treatment and placebo-controlled groups, respectively. All bevacizumab-treated patients showed improvement in clinical neurological symptoms and signs [5]. Thus, it has been well established that bevacizumab is capable of improving overall neurological signs and symptoms in patients suffering from symptomatic radiation necrosis.

Despite these positive findings, further study of the effects of bevacizumab on patients of different populations is merited due to the remaining controversy regarding the efficacy and risks involved with bevacizumab treatment. An analysis of over 40 studies on bevacizumab treatment for radiation necrosis indicate that the majority of studies report positive findings [14], with bevacizumab thought to play a beneficial role in restoration of the blood–brain barrier, some studies have suggested that bevacizumab may actually have detrimental effects. One such study suggests that while bevacizumab may have initially beneficial results, it may soon cause over-pruning of affected blood vessels, resulting in vascular insufficiency that can exacerbate hypoxia and tissue necrosis [7]. Thus, the current study provides significant evidence to support previous findings indicating that bevacizumab treatment does not produce these additional risks in Chinese cerebral radiation necrosis patients.

The current study suggests that bevacizumab treatment was very well tolerated, with no findings of bevacizumab-related toxicity greater than grade III. Serious adverse events previously associated with bevacizumab treatment, such as cerebral hemorrhage, gastrointestinal perforation and arterial thromboembolic events were not observed in this group of patients. This may be attributed to careful screening of patients for concurrent conditions, potentially involved in the onset of these severe effects, prior to inclusion. Notably, the current study also demonstrated that bevacizumab decreases perilesional edema caused by radiation necrosis (Figure 1), and 16 of 17 patients achieved a significant clinical improvement in neurologic symptoms caused by radiation necrosis. Unfortunately, three patients experienced only a short duration of symptom relief during the first bevacizumab treatment; however, four patients were able to completely discontinue steroid use after the second bevacizumab injection. Only a single patient responded poorly to bevacizumab treatment, with a mild response indicated even in this case based on the achievement of lowered dexamethasone dosage. Because all patients remained on dexamethasone prior to bevacizumab treatment, a delayed steroid-associated effect cannot be excluded. Therefore, further studies will be required to confirm these findings.

Although rapid relief of radiation necrosis was achieved in the majority of cases examined in the current study, seven patients demonstrated aggravated clinical status over a period of several months after cessation of bevacizumab therapy, wherein perifocal edemas reappeared and enlarged. Dynamic gadolinium-enhanced and T2-weighted MRI images and MRS studies revealed five cases of tumor recurrence and another two cases of radiation necrosis. No attempt was made to administer secondary bevacizumab treatment to these patients, though the effects of such secondary treatment merit further investigation. Monomasa *et al.* recently reported that secondary bevacizumab treatment resulted in improved efficacy in controlling both radiation necrosis and edema. This treatment also improved clinical symptoms at levels similar to those observed in the initial round of treatment, though the effect on future relapse remained undocumented [15]. The benefits of secondary bevacizumab treatment clearly provided at least temporary relief of symptoms, suggesting that secondary bevacizumab treatment should be recommended in cases of recurrence of radiation necrosis and edema.

The recurrence of radiation necrosis after bevacizumab treatment raises an important issue about the optimization of drug dosages and treatment intervals. Though four bevacizumab doses (7.5 mg/kg) have been reported to be sufficient for attaining long-term resolution for most patients, recent studies have revealed that a single round of multiple bevacizumab treatments might be unable to achieve long-term resolution. Levin *et al.*[5] reported that two patients previously treated with four doses of bevacizumab required one to two additional doses to reestablish necrosis reduction at 36 to 38 weeks after initial bevacizumab treatment. Similarly, Motomasa *et al.*[15] also reported two patients with recurrent radiation necrosis several months after initial biweekly bevacizumab treatment (5 mg) of six injections. These studies provide strong evidence that future trials will be required to validate the most effective treatment regimens.

## Conclusions

The current study demonstrates that bevacizumab treatment shows great potential in treating cerebral radiation necrosis caused by common forms of radiation therapy, including EBRT, SRS and FSRT. While controversy still exists as to both the efficacy and risk associated with bevacizumab treatment in cerebral radiation necrosis patients, the current findings demonstrate bevacizumab’s impressive overall safety profile. These results confirm previous positive findings for the ability of bevacizumab treatment to reverse radiation necrosis, suggesting that previous studies evidencing exacerbation of radiation necrosis following bevacizumab treatment may have resulted from the presence of complicating conditions, eliminated by careful screening of the patients included in the present study. Future studies will be necessary in order to achieve longer, more consistent improvement or alleviation of neurologic symptoms, cerebral radiation necrosis, and edema through optimization of treatment regimens and strategies.

## Abbreviations

AVM: arteriovenous malformation; EBRT: external beam radiotherapy; FDG-PET: ^18^F-2-fluro-D-deoxy-glucose-positron emission tomography; FLAIR: fluid attenuated inversion recovery; FSRT: fractionated stereotactic radiotherapy; KPS: Karnofsky performance status; MRI: magnetic resonance imaging; MRS: magnetic resonance spectroscopy; RT: radiation treatment; SRS: stereotactic radiosurgery; T2W: T2-weighted MRI; VEGF: vascular endothelial growth factor.

## Competing interests

The authors declare that they have no competing interests.

## Authors’ contributions

YW, LP, XS, JD, EW, and NZ designed the study, collected the case information, drafted the manuscript, and performed statistical analysis. YM and YY designed the study and collected the case information. All authors read and approved the final manuscript.
